# Synovial Sarcoma of the Hand: A Case Report and Literature Review on a Significant Challenge in Treatment

**DOI:** 10.7759/cureus.79898

**Published:** 2025-03-01

**Authors:** Syeda Sara Tajammul, Khadiga Mohammed, Humaid Al Farii, Asem Shalaby, Javeria Munir, Iqbal Al-Amri, Mahmoud Alfishawy, Zahid Al Mandhari

**Affiliations:** 1 Radiation Oncology, Sultan Qaboos Comprehensive Cancer Care and Research Centre (SQCCCRC), Muscat, OMN; 2 Radiation Oncology, University Medical City, Muscat, OMN; 3 Orthopedics, Sultan Qaboos University Hospital, Muscat, OMN; 4 Orthopedics, University Medical City, Muscat, OMN; 5 Pathology, Sultan Qaboos University Hospital, Muscat, OMN; 6 Pathology, University Medical City, Muscat, OMN; 7 Pathology, College of Medicine, Mansoura University, Mansoura, EGY; 8 Radiology, Sultan Qaboos Comprehensive Cancer Care and Research Centre (SQCCCRC), Muscat, OMN; 9 Radiology, University Medical City, Muscat, OMN

**Keywords:** extremity, hand, soft tissue sarcoma (sts), synovial sarcoma, upper limb

## Abstract

Soft tissue sarcomas (STS) of the hand are extremely uncommon, with synovial sarcomas being even rarer. Despite their small size at presentation, hand sarcomas can indeed be aggressive and may result in significant morbidity and mortality if not effectively managed. A considerable proportion of these tumors are treated by unplanned excision before referral to a specialist oncological center. Additionally, hand sarcomas can mimic benign lesions, leading to misinterpretation and delayed diagnosis. To address these challenges, it is essential to thoroughly investigate all atypical soft tissue masses arising in the hand. Surgery is indeed considered the primary treatment for STS of the hand. However, achieving wide surgical margins can be particularly challenging in this area due to the intricate anatomy and proximity of tumors to vital structures within the hand. Adjuvant radiotherapy plays a crucial role in achieving local tumor control by targeting microscopic tumor cells near the surgical bed. This approach allows for a planned close margin excision along critical structures, which is essential for reducing the risk of tumor recurrence while preserving hand function. The role of adjuvant chemotherapy for synovial sarcoma of the hand is controversial and still a matter of intense debate. Distant metastasis and local recurrence are common in synovial sarcoma cases. They mostly metastasize to the lungs, followed by lymph nodes, bones, and the liver.

Multidisciplinary discussions involving surgeons, medical oncologists, radiation oncologists, and other specialists are crucial in making informed decisions about the most appropriate treatment approach for each individual patient with STS of the hand. This collaborative approach ensures that the chosen treatment plan optimizes both oncological outcomes and the patient's overall quality of life.

## Introduction

Soft tissue sarcomas (STS) of the hand are rare entities, compromising 1% of all STS cases; synovial sarcoma of the hand is even rarer [1]. For hand STS, surgery is thought to be the main course of treatment [2]. However, because of the complex anatomy and proximity of tumors to important tissues within the hand, achieving wide surgical margins can be extremely difficult in this area [3]. As a result, surgeons should strike a careful balance between preserving the hand function and obtaining oncologic clearance. Maintaining this equilibrium needs meticulous preoperative planning, which includes detailed imaging evaluation and consideration of various surgical techniques [4]. Adjuvant radiation may be considered for limb salvage and to help lower the risk of local recurrence in situations where achieving wide surgical margins is impossible [5].

We report a case of synovial sarcoma of the hand in a 28-year-old female patient who was diagnosed by a histological examination of the excised mass. We discuss the characteristics of synovial sarcoma on magnetic resonance images and histopathology. Literature is specifically reviewed about the management of the disease.

## Case presentation

**Figure 1:**
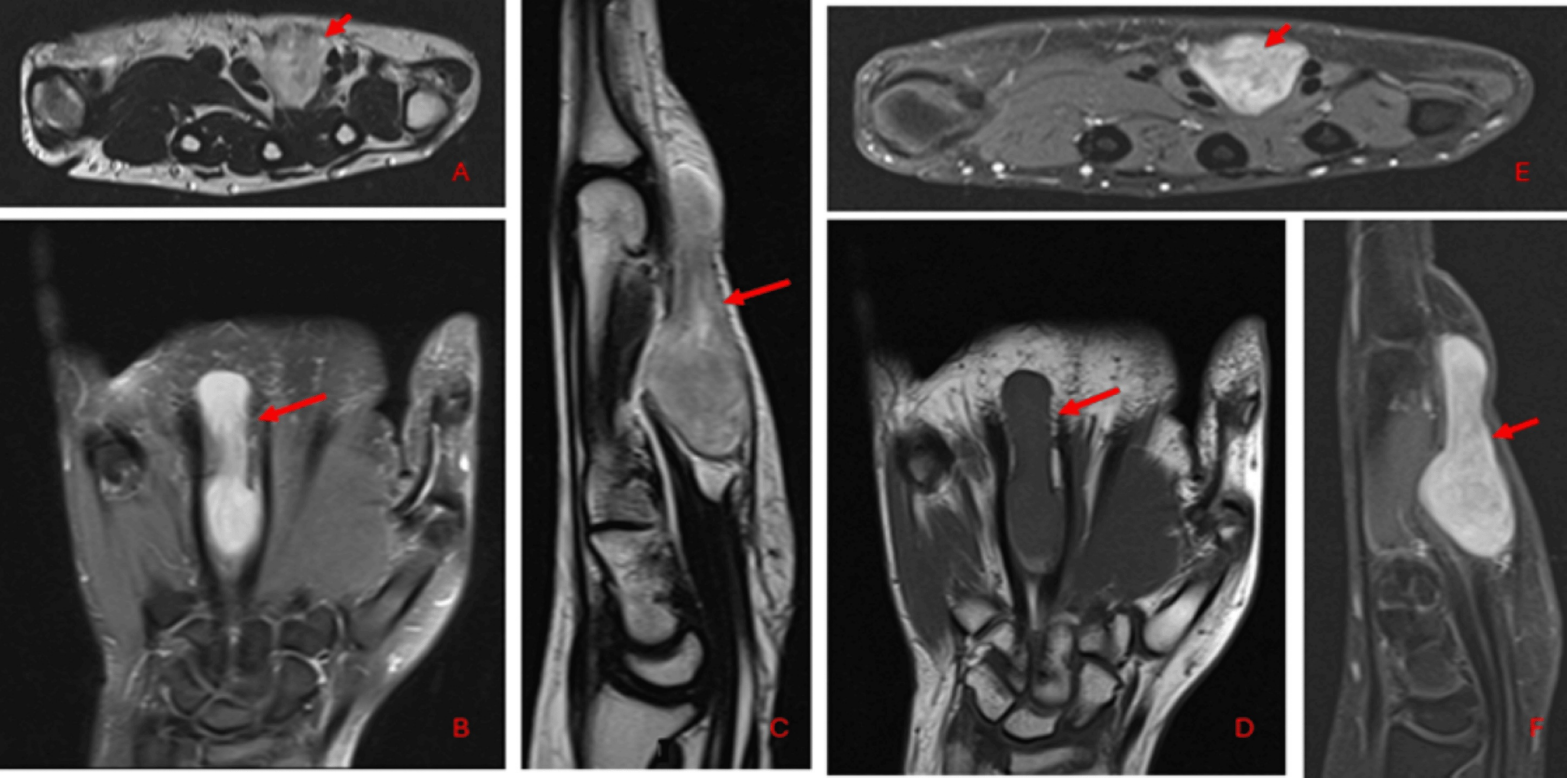
Preoperative MRI of the hand showing a lobulated mass within the intercarpal space between the third and fourth flexor tendons and lumbricals. It demonstrates hyperintense signals (red arrows) on axial T2WI (A), coronal PD (B), and sagittal T2WI (C) images and hypointense signals on coronal T1WI images (D). Intense enhancement on axial (E) and sagittal (F) post-IV gadolinium contrast images are also observed MRI: magnetic resonance imaging; T2WI: T2-weighted imaging; PD: proton density; T1WI: T1-weighted imaging; IV: intravenous

We present a case of a 28-year-old female patient with no known comorbidities who presented at a private hospital with a four-month history of a lump in her left hand that progressively increased in size. MRI of the hand showed a lobulated mass within the intercarpal space between the third and fourth flexor tendons and lumbricals (Figure 1). CT scan of the chest, abdomen, and pelvis (CAP) was negative for distant metastasis.

**Figure 2:**
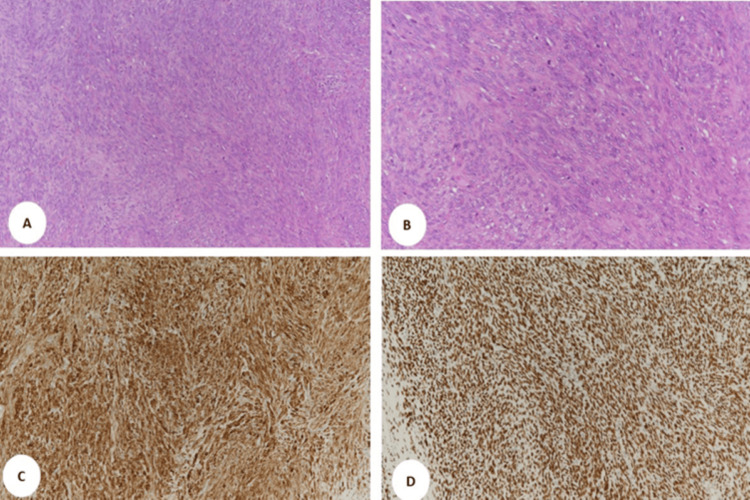
Photomicrograph of histologic section from the case with H&E stain at magnifications of (A) 100× and (B) 200×, showing spindle cell proliferation with evident mitotic figures. (C) Immunohistochemical staining for BCL2 antibody (100×) showing diffuse strong positive membrane and cytoplasmic immune reactivity. (D) Immunohistochemical staining for TLE1 antibody (100×) showing diffuse strong positive nuclear immune reactivity H&E: hematoxylin and eosin

She underwent a wide local excision of the lesion, and histopathology showed monophasic synovial sarcoma. The tumor size was 4.7 cm. In grade 2, lymphovascular space invasion and perineural invasion were negative, and tumor cells were seen focally at inked margins. Ki 67 15%-20%, pT1NxMx (pTNM, American Joint Committee on Cancer 8th edition) immunohistochemistry (IHC) showed diffuse positivity for TLE1, BCL2, CD99, FLT1, CD56, Histone 3, and Calponin (Figure 2). Fluorescence in situ hybridization is positive for SS18 gene rearrangement, which supports the diagnosis of synovial sarcoma.

**Figure 3:**
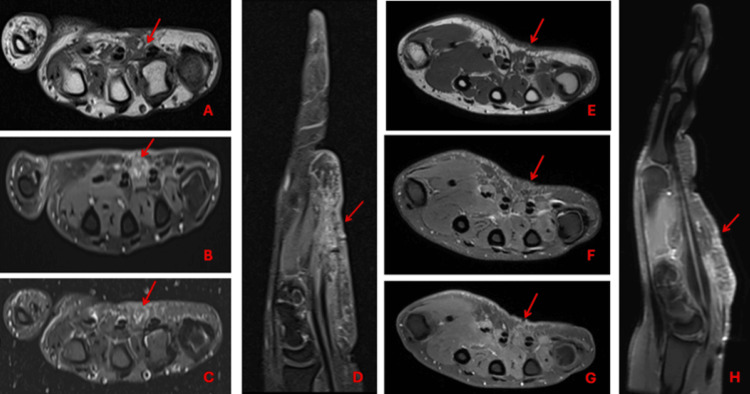
Postoperative MRI of the hand done at one month showing postsurgical changes (red arrows) on axial T1 (A), post-gadolinium-enhanced axial T1 fat-suppressed (B), axial T2 fat-suppressed (C), and sagittal PD fat sat (D) images. Postoperative MRI of the hand done at three months showing postsurgical changes and scarring (red arrows) on the axial T1 fat-suppressed (E), axial PD fat-suppressed (F), post-gadolinium-enhanced axial (G), and sagittal (H) images MRI: magnetic resonance imaging; PD: proton density

Post-op MR of the left hand done after one month shows acute post-op changes rather than residual disease (Figure 3). The case was discussed in the multidisciplinary meeting (MDT), and she was planned for adjuvant radiotherapy followed by close surveillance with MR hand and CT chest. The option of re-resection/hand amputation was also discussed but not recommended because of functional morbidities.

**Figure 4:**
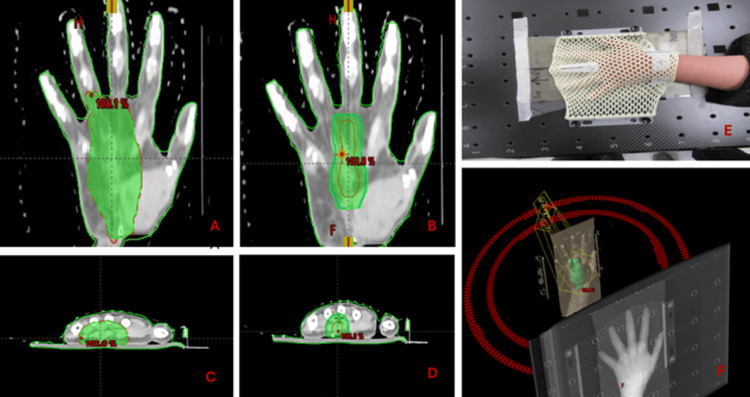
Radiotherapy treatment planning including coronal and axial images showing targets and the radiation dose cloud at different dose levels of 50 Gy (A,C) and 60 Gy (B,D). Patient hand immobilization in a prone position with a thermoplastic mask (E). A 3D representation of the room eye view of the patient hand and beam geometry by the VMAT technique (F) VMAT: volumetric arc therapy

**Figure 5:**
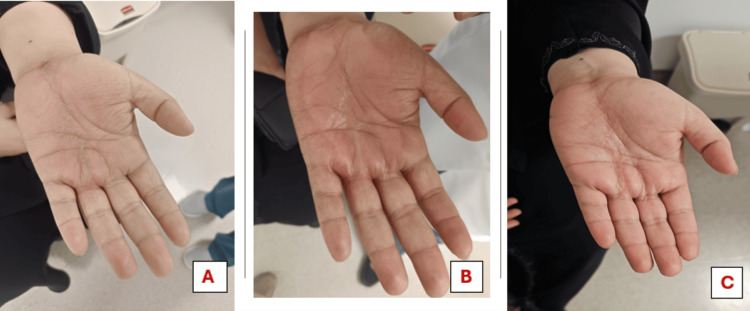
Images of the left hand during and after radiotherapy. No radiation dermatitis noted after completion of seven fractions (A). Grade 2 dermatitis with moist desquamation, redness, tenderness, and edema of the left hand noted after completing 15 fractions (B). Mild edema, redness, and tenderness are still present at three months post radiotherapy (C)

The patient was then referred to the radiation oncology department for adjuvant radiation. She was initially planned for adjuvant radiotherapy 66 Gy in 33 fractions in two phases (Figure 4). After completing 15 fractions of phase 1 (pre-op gross tumor volume, GTV, +1 cm margin for clinical target volume, and +5 mm margin for planning target volume, PTV), she developed swelling, redness, and tenderness in her hand, which resulted in her treatment being withheld for 5 days. She was also started on oral steroids. Her case was discussed again during the peer review in the radiation oncology department. Due to the risk of developing compartment syndrome, which could result in the amputation of her hand, the consensus was to shift her to phase 2 (pre-op GTV +5 mm margins for PTV). It was also decided to reduce the total radiation dose to 60 Gy in 30 fractions rather than 66 Gy in 33 fractions. After three months following the completion of radiotherapy, the patient still exhibits mild redness, tenderness, and swelling of the left hand, while movements of the hands remain normal. She was advised to undergo hand physiotherapy. An MRI of the left hand at three months (Figure 5) showed postsurgical changes with mild edema and scarring but no evidence of recurrent disease.

## Discussion

Synovial sarcomas are rare malignant tumors, representing 5%-10% of STS [6]. Despite their name, synovial sarcomas do not actually originate from synovial tissue. Instead, they usually occur near joint spaces, which is the source of the misnomer [7]. Synovial sarcomas have a predilection for adolescents and young adults, typically occurring between the ages of 10 and 40 [8]. While there is a slight male preponderance, this tumor can affect individuals of all ethnic backgrounds [9]. Although these tumors can arise in any location throughout the body, they are mostly found in the extremities, particularly around the large joints like the knee [10].

STS of the hand is exceedingly rare and comprises 1% of all upper limb tumors. Synovial sarcoma is even rarer [1]. In the studies mentioned so far, the most common histological subtype in this region includes malignant fibrous histiocytoma, followed by liposarcoma [1]. As noted in our case, synovial sarcoma of the hand most often arises on the palmer aspect of the hand, followed by the fingers [3].

STS of the hand pose unique management challenges, first due to their intricate anatomical structures. This region has limited soft tissue with critical structures tightly packed together. The proximity of important structures such as nerves, blood vessels, tendons, and bones increases the risk of severe functional deficit [11]. Second, the scarcity of comprehensive data about the behavior of STS of the hand is a significant challenge in developing specific standardized treatment protocols. Many existing studies in this field rely on individual case reports, which offer limited evidence due to their anecdotal nature and small sample sizes [12].

STS of the hand are often small at presentation. This can lead to earlier detection compared to sarcomas in other body parts. However, despite their small size, hand sarcomas can indeed be aggressive and may result in significant morbidity and mortality if not effectively managed [13]. A significant proportion of these tumors (38%-95%) are treated by unplanned excision before referral to a specialist oncological center [14]. The challenges in diagnosing these tumors often stem from their small size, which may not meet the conventional criteria of 5 cm for referral to a specialized sarcoma MDT [15]. Additionally, hand sarcomas can mimic benign lesions such as ganglion cysts or glomus tumors, leading to misinterpretation and delayed diagnosis [16]. To address these challenges, it is essential to thoroughly investigate all atypical soft tissue masses arising in the hand. This typically involves a comprehensive diagnostic workup that includes a core biopsy and an MRI scan. If there is a suspicion of a sarcoma diagnosis, a biopsy should be performed by a surgeon on the soft tissue tumor MDT, and ideally, the same person should perform the definitive resection. CT CAP should be performed to rule out distant metastasis once the diagnosis of STS has been set up [13].

Histologically, synovial sarcoma can present with three main variants: monophasic, biphasic, and poorly differentiated. As the name indicates, the monophasic variant of synovial sarcoma consists of one cellular component, typically spindle cell morphology. In contrast, the biphasic variant includes both spindle cell and epithelial cell components. Poorly differentiated synovial sarcoma is characterized by hypercellularity, hyperchromatic nuclei, polymorphism, and increased mitotic activity. Necrosis is commonly seen in this variant [17].

A characteristic cytogenetic abnormality found in all cases of synovial sarcoma is the translocation t(X;18) (p11.2; q11.2). This abnormality helps provide diagnostic confirmation and is a valuable tool in distinguishing synovial sarcoma from other soft tissue tumors [17]. Synovial sarcomas are strongly positive for TLE1 on IHC, while other sarcomas and carcinomas can rarely express TLE1. Strong and diffuse staining for TLE1 is often considered a characteristic feature of synovial sarcomas [18].

Surgery is indeed considered the primary treatment for STS of the hand [2]. However, achieving wide surgical margins can be particularly challenging in this area due to the intricate anatomy and proximity of tumors to vital structures within the hand [3]. There is substantial uncertainty regarding safe surgical margins for treating forearm and hand sarcomas [6].

Traditionally, the management of STS in the hand often involved radical resection, which sometimes required amputation to ensure complete removal of the tumor. However, recent studies have shown that conservative resection, where only the tumor and minimal surrounding tissue are removed, combined with radiation therapy, can yield comparable long-term survival outcomes [19]. The study by Talbert et al. highlighted the lack of a survival benefit from amputation and advocated for limb-sparing strategies. These findings have led to a shift in treatment strategy toward limb salvage procedures, with the goal of preserving hand function and esthetics whenever possible [20]. Surgeons may employ compartmental resection, microsurgical reconstruction, and nerve grafting techniques to achieve the best possible outcome. In cases where achieving wide surgical margins is impossible, adjuvant radiotherapy may help reduce the risk of local recurrence. Despite the shift toward limb salvage procedures, the decision between limb salvage and amputation stays complex and must be individualized for each patient. In situations where the tumor is large and aggressive or involves critical structures to the extent that functional preservation is not possible, amputation may still be necessary to ensure complete tumor removal and prevent recurrence [4].

Adjuvant radiotherapy is crucial in achieving local tumor control by targeting microscopic tumor cells near the surgical bed. This approach allows for a planned close margin excision along critical structures, which is essential for reducing the risk of tumor recurrence while preserving hand function [5]. In the randomized prospective study on adjuvant radiotherapy, local recurrence occurred in only 1.4% of patients who received adjuvant radiation therapy, compared to a much higher rate of 23.9% in patients who did not receive radiation therapy, irrespective of the type of margin [21]. However, the use of radiotherapy in this context is not without its challenges and potential early and late complications, including wound dehiscence, wound necrosis, fibrosis, and ankylosis. Joint contracture, adduction contracture, osteitis, and potential for radiation-associated fractures have been reported in view of the low radiation tolerance of the skin of the palm [22].

The advancement in radiotherapy techniques has contributed to improved outcomes for patients with hand STS. This includes improved targeting by recognition of the importance of excluding the entire joint from the high-dose volume and sparing a corridor of normal tissue, advanced imaging by the routine use of CT and MRI scans to define tumor volume, and improved immobilization techniques that help ensure consistent positioning of the patient during radiotherapy sessions. These techniques have led to more precise tumor targeting while minimizing radiation exposure to adjacent healthy tissues and critical structures [23].

Various modalities of adjuvant radiation therapy can be used, including brachytherapy, electron therapy, three-dimensional conformal radiation therapy, intensity-modulated radiation therapy, and volumetric arc therapy. Each modality offers unique advantages and may be tailored to the individual patient's needs and tumor characteristics [24]. Studies have shown that, regardless of the type of radiation therapy used, employing radiation therapy leads to higher local control rates in patients with synovial sarcoma [25].

The optimal radiation therapy dose for synovial sarcoma of the hand remains a topic of debate, with inconsistent recommendations across different international guidelines. However, studies have suggested that a dose of around 61.2 Gy (with a range typically between 45 and 66.6 Gy) may be sufficient for adjuvant radiotherapy. This dose has been associated with favorable five-year and 10-year overall survival rates [26]. The timing of radiotherapy in relation to surgery is another important consideration. While both preoperative and postoperative radiotherapy have been shown to achieve similar levels of local control and long-term functional outcome, the postoperative wound complication rates are higher with preoperative radiotherapy [25]. The role of adjuvant chemotherapy for synovial sarcoma of the hand is controversial and still a matter of intense debate [27].

Distant metastasis and local recurrence are common in synovial sarcoma cases. They mostly metastasize to the lungs (80%), followed by lymph nodes (15%-20%), bones (9.9%), and the liver (4.5%) [28,29]. Synovial sarcoma is thought to have a five-year survival rate between 27% and 55%. Research revealed that the 10-year survival rate for distal tumors, which include the hand and foot, was higher than that of proximal extremities (65% vs. 48%) [30].

## Conclusions

Synovial sarcoma of the hand is a rare condition that poses unique challenges in management: first due to its intricate anatomy and second due to the lack of development of specific treatment protocols. In this regard, multidisciplinary discussions involving surgeons, medical oncologists, radiation oncologists, and other specialists are crucial in making informed decisions about the most appropriate treatment approach for each individual patient with STS of the hand. This collaborative approach ensures that the chosen treatment plan optimizes both oncologic outcomes and the patient's overall quality of life.
